# Factors Associated With Corneal Ulceration in Horses: 420 Cases (2020–2023)

**DOI:** 10.1111/vec.70149

**Published:** 2026-08-14

**Authors:** Valeria Adamchick Galindo, Langdon Fielding

**Affiliations:** ^1^ Loomis Basin Equine Medical Center Penryn California USA

**Keywords:** blindness, enucleation, eye, trauma, weather

## Abstract

**Objective:**

To evaluate how environmental and other risk factors are associated with the prevalence of corneal ulceration in horses presenting to equine emergency veterinarians in the Western United States.

**Design:**

Retrospective study conducted from May 2020 to November 2023.

**Setting:**

Equine field and hospital practice in the Western United States.

**Animals:**

Four hundred twenty horses with corneal ulceration, confirmed by clinical examination and fluorescein staining, of any underlying cause.

**Interventions:**

None.

**Measurements and Main Results:**

The primary study outcomes were the association between environmental factors (wind speed, temperature, dew point) and corneal ulceration, as well as factors associated with the need for enucleation. Using a Poisson regression model, increased average wind speed (*p* < 0.01) and increased average temperature (*p* = 0.08) were associated with higher rates of corneal ulceration. Male horses were more likely to develop ulcers (*p* = 0.004) compared with females. Older age (*p* < 0.0001), deeper ulcers (*p* < 0.0001), and involvement of the left eye (*p* < 0.05) were associated with an increased risk for enucleation. The presence of uveitis was also associated with the need for enucleation (*p* = 0.002).

**Conclusions:**

Equine emergency veterinarians should be aware that both wind speed and increasing temperatures are associated with corneal ulceration in the Western United States. Enucleation in horses with corneal ulcers is associated with increasing age, uveitis, and increasing depth of the ulcer.

**Abbreviations**
AIC: Akaike information criterion

## 1 Introduction

Many forms of ocular disease are considered emergencies by equine clients and veterinarians, and ophthalmic cases represent 6% of all equine emergencies [1]. Corneal ulceration is one of the more common ocular diseases, and equine emergency veterinarians should be familiar with its risk factors, diagnosis, and treatment [2, 3]. The large size of the equine eye, the laterally prominent position of the globe, natural behavior of the horse, and physical activity may all predispose horses to the development of corneal ulcers [4]. These factors lead to an exposed cornea that is vulnerable to trauma and toxic or infectious diseases [5].

Retrospective studies of equine corneal disease have primarily focused on deep stromal abscesses or keratomycosis [6, 7, 8]. Increased wind speed was associated with a greater prevalence of these more serious conditions, but a rigorous analysis of environmental factors associated with corneal ulceration in horses presenting to equine veterinarians does not exist [6, 7]. One study in Thoroughbred racehorses identified an association with age, showing that 2‐year‐old horses were more likely to be diagnosed with corneal ulceration; however, a wide age range was not represented in the study, and racehorses have some unique and specific causes of this condition [9]. A retrospective study of horses presenting with ulcerative keratitis reported a 77% incidence of surgical treatment of the ulcer, suggesting an atypical case presentation compared with more standard emergency practice [10].

The purpose of this retrospective study was to evaluate the association of environmental and other risk factors with the prevalence of corneal ulceration in horses presenting to equine emergency veterinarians. We hypothesized that environmental factors such as wind speed would be associated with an increased risk of corneal ulceration, similar to the more serious conditions of stromal abscess and ulcerative keratomycosis. Additionally, the study describes the signalment and clinical findings of horses with corneal ulceration and the population of horses that require enucleation. Overall, the goal of this paper was to provide equine emergency veterinarians with findings that guide diagnosis and treatment when evaluating equine corneal ulcers.

## 2 Materials and Methods

### 2.1 Criteria for Case Selection

Using the key words “ulcer” and “cornea,” the medical records of the Loomis Basin Equine Medical Center were searched for corneal ulcer cases presenting between May 2020 and November 2023. Cases were included if the medical records indicated a diagnosis of corneal ulceration that was made by clinical examination and a positive fluorescein staining of the cornea. Cases could have been subsequently evaluated by an ophthalmologist, but the first evaluation was performed by veterinarians without board certification in ophthalmology.

Information retrieved from the medical records included signalment, laterality (left, right, or both eyes) of the affected eye, additional ophthalmologic findings, and date of initial diagnosis. Based on the medical records, cases were categorized into the following groups: trauma (e.g., presence of non‐ocular clinical signs, such as head trauma), visual impairment (e.g., blind in one or both eyes), neurologic (typically facial nerve paralysis), uveitis, foreign body, allergic, and neoplasia. If cases were not associated with one of these descriptions, they were categorized as “other.”

Corneal ulcers were recorded as deep, superficial, corneal perforation, or indolent‐type ulceration. The classification of indolent‐type (nonhealing) ulceration was based on statements made in the medical record. In addition to these data, treatment and resolution of the case (medical resolution, enucleation, or euthanasia) were recorded. For each case that was euthanized, the reason for euthanasia was assessed from the medical record to determine whether it was financial, specifically for ocular disease, or for other comorbidities (e.g., head trauma with an associated corneal ulcer). Horses were categorized as “enucleation” if the procedure was performed or recommended but euthanasia was chosen instead. The reason for enucleation was also determined from the medical record.

For each month during the study period, historical weather, including maximum high temperature, minimum low temperature, average monthly temperature, average wind speed, and average dew point, was collected from a weather database [11].

### 2.2 Statistical Analysis

Data are reported as mean ± SD for normally distributed data and median (range) for nonnormally distributed data. Normality was determined using the Kolmogorov–Smirnov test.

A Poisson regression model was used to determine the effects of weather variables on the frequency of corneal ulceration. An initial univariate analysis was completed for each weather parameter; variables with a *p*‐value of <0.10 were included in the final analysis. Minimizing Akaike information criterion (AIC) in a forward stepwise fashion was used as the basis for the final selection model. Variables with a *p*‐value of <0.10 were accepted in the final model if their addition improved the overall AIC.

The age, sex, and categorization of horses with corneal ulcers requiring enucleation were compared with horses that did not require enucleation. Continuous variables were assessed using a *t*‐test or Mann–Whitney *U*‐test, while Fisher's exact test was used for categorical variables. A commercially available software package was used for the statistical analysis^1^, with *p* < 0.05 considered statistically significant.

## 3 Results

A total of 420 cases met the inclusion criteria. Table 1 shows the characteristics for all horses with corneal ulceration. Within the study group, male horses were overrepresented compared with females (*p* = 0.004). There was no difference in laterality of the ulceration. Two hundred seventy‐four of 420 horses (65%) had an unknown cause for their ulceration, while the cause was allergy‐based in 41 horses (10%), uveitis in 30 (7%), trauma in 22 (5%), visual impairment in 21 (5%), foreign body in 19 (5%), neurologic in 9 (2%), and neoplasia in 4 (1%). No horses had ocular surgery other than enucleation, and ulcers were therefore all treated medically.

**TABLE 1:** Characteristics (age, sex, breed, and affected eye) of 420 horses presenting with corneal ulceration in the Western United States (2020–2023).

Variable	Results	Number of horses (*n*)	*p*‐value
Sex		397	0.004
Male (% of total)	57.18	227	
Female (% of total)	42.82	170	
Age (years)	15 ± 9	396	
Breed			
Quarter Horse		71	
Arabians		36	
Thoroughbred		35	
Miniature		24	
Other		231	
Laterality		411	0.72
Right eye (%)	48	200	
Left eye (%)	46	193	
Both eyes (%)	4.38	18	

*Note*: Data are presented as mean ± SD.

The results of univariate analysis for the Poisson regression model are reported in Table 2; the final Poisson model is shown in Table 3. An increase in the monthly average wind speed (*p* < 0.01) and an increase in monthly average temperature (*p* = 0.08) were both associated with greater frequency of corneal ulceration.

**TABLE 2:** Univariate analysis for Poisson regression evaluating the association between monthly weather data and the monthly frequency of corneal ulceration in 420 horses in the Western United States.

Variable	Estimate	*p*‐value
Maximum monthly temperature (°C)	0.015	0.01
Average monthly temperature (°C)	0.023	<0.01
Minimum monthly temperature (°C)	0.024	0.01
Total monthly precipitation (cm)	−0.016	0.8
Average monthly wind speed (km/h)	0.07	<0.001
Average dewpoint (°C)	0.033	0.02

*Note*: Positive estimates indicate increased ulcer frequency with increasing values of the variable. Average monthly wind speed and temperature were associated with ulcer frequency.

**TABLE 3:** Final multivariate Poisson regression model evaluating the association between monthly weather data and the monthly frequency of corneal ulceration in 420 horses in the Western United States.

Variable	Estimate	95% CI	*p*‐value
Average monthly temperature (°C)	0.015	−0.0016 to 0.0315	0.08
Average monthly wind speed (km/h)	0.058	0.0158 to 0.1017	<0.01

*Note*: Average monthly temperature and average monthly wind speed were both associated with increased frequency of corneal ulceration.

Abbreviation: CI, confidence interval.

Table 4 compares the horses with enucleation with those that were resolved medically. Enucleation was performed in 26 of 420 (6%) horses with corneal ulcers, and 7 (2%) were euthanized due to ocular disease, for which enucleation was declined despite recommendation. An additional six horses were euthanized for reasons apparently unrelated to the corneal ulceration. When listed, reasons for enucleation or euthanasia (due to ocular disease) included ruptured cornea (*n* = 6), lack of response to medical treatment (*n* = 8), behavioral reasons making treatment impossible (*n* = 3), concurrent ocular disease in addition to the ulcer (*n* = 13), deep ulceration (*n* = 9), financial considerations (*n* = 1), and advanced age (*n* = 1). Each incidence of corneal ulceration could have more than one reason for enucleation.

**TABLE 4:** Comparison of horses with corneal ulceration that underwent enucleation (or recommended enucleation) with those that did not, among 420 horses in the Western United States with corneal ulceration.

Variable	Enucleated or euthanized for ocular reasons	Not enucleated	Odds ratio (95% CI)	*p*‐value
Age (years)	22 ± 6	15 (0−39)	N/A	<0.0001
Male	18/32	209/387	1.095 (0.5444–2.316)	0.86
Left eye	23/33	197/387	0.451 (0.2199–0.9711)	<0.05
Cause	Cases (*n*)	Cases (*n*)		
Allergic	3	37	0.946 (0.2913–3.150)	0.99
Trauma	0	22	0.00 (0.000–1.865)	0.24
Neurologic	1	8	1.48 (0.1296–10.31)	0.52
Foreign body	0	19	0.00 (0.000–2.225)	0.38
Uveitis	9	21	6.536 (2.726–15.32)	0.0002
Vision impairment	2	19	1.25 (0.2768–4.732)	0.68
Neoplasia	1	3	4.0 (0.2993–27.32)	0.28
Other	17	258	0.53 (0.2696–1.064)	0.09
Ulcer description				
Superficial	7	324	0.0524 (0.02064–0.1222)	<0.0001
Deep	15	29	10.29 (4.825–23.00)	<0.0001
Nonhealing	2	29	0.796 (0.1800–3.155)	0.99
Corneal rupture	7	0		N/A

*Note*: Data are presented as mean ± SD or median (range).

Abbreviations: CI, confidence interval; N/A, not applicable.

## 4 Discussion

In the current study, sex, increasing monthly temperature, and increasing monthly wind speed were found to be risk factors for the development of corneal ulceration in emergency equine practice. Horses that underwent enucleation or euthanasia for ocular reasons were more likely to be older, have deeper corneal ulcers, and have involvement of the left eye.

More male horses are represented in this study population (57.18%) than females (42.82%). This was consistent with a previous study from Japan that identified stallions and 2‐year‐old horses as more frequently affected and prone to develop ulcerative keratitis [9]. With a study population of 2‐ to 5‐year‐old racehorses, the age composition in that study was highly biased toward young horses [9]. The study from Japan also evaluated stallions, whereas our study had very few stallions. In a study from Florida, 68% of the horses with corneal ulcers were male [12]. Corneal ulcers may be more common in male horses due to the animals’ different uses (e.g., geldings are more likely to be used for riding as opposed to brood mares). Additionally, male horses might be more likely to play or fight. In people, many ocular diseases are more prevalent in women, but corneal ulceration has been described more commonly in men [13, 14].

In the final Poisson regression model, increasing monthly temperature and increasing wind speed were associated with a greater incidence of corneal ulceration. A study from North Central Florida identified wind speed as a risk factor in horses, with increases leading to a more frequent incidence of deep stromal abscesses [6]. Environmental microtrauma from wind may induce abnormalities of tear film, or accelerated particulate matter in the air may cause epithelial damage [6]. A study from Colorado evaluating environmental factors associated with ulcerative keratomycosis found a similar correlation between higher wind speed and increased risk for ulcerative keratomycosis. The overall prevalence was highest in the summer and fall [7]. The statistical model used in our study identified higher monthly temperatures, not just the overall seasonality of summer, as a risk factor for corneal ulceration.

An association between corneal ulcers and environmental factors can help equine veterinarians understand the important risk factors. Recognizing that hot and windy conditions increase the risk for corneal ulceration could help educate clients about times when it may be more important to provide ocular protection. These findings suggest that these environmental variables are risk factors not just for abscesses and ulcerative keratomycosis but also for uncomplicated corneal ulceration.

In the current study, the median age of horses with corneal ulcers was 15 ± 9 years. In a retrospective study of equine corneal ulcers from Germany, the average age of patients with corneal ulcers was significantly higher than the reference case population, with a mean age of 13.7 years [10]. In another study from Belgium, the mean age of horses with corneal ulceration was 12 years [15]. Age‐related predisposition for the development of corneal ulceration in older horses may be associated with changes in the anatomic structure, physiology, and immunology of the older cornea [10]. Previous literature in dogs determined that each additional year of age increased the odds of corneal ulcers by 8.9% [16]. Older age has also been associated with an increased risk of corneal ulceration in people [12].

The association between both age and ulcer depth with enucleation was not surprising. As horses age, they may have other comorbidities that make management more difficult. Also, elder horses’ behavior and use may render monocular vision adequate when they are older, thus allowing for this treatment option. A prospective study would be needed to better identify the factors contributing to the decision for enucleation in an older horse.

An increased risk factor for enucleation of the left eye was seen in the current study, a finding that has not been observed in people [17]. The difference was marked, with 70% of enucleations in this study being performed (or recommended) due to involvement of the left eye. Our practice has a minimal racehorse population; therefore, an association with direction of work is unlikely. This finding was surprising and should be evaluated in future research projects.

The most common reason leading to euthanasia or enucleation was concurrent eye disease and, particularly, uveitis. Other reasons included patients not responding to treatment, deep or ruptured ulcers, behavioral issues preventing appropriate treatment, and financial/age concerns. In some cases, more than one reason was given (e.g., uveitis and poor response to treatment). It is important to recognize that many of these reasons were not medical. In a retrospective study on nonhealing ulcers, the most common cause for enucleation was uncontrollable keratomycosis [18]. The current study would have been stronger if cytology results were available for analysis.

The current study has a number of limitations common to retrospective evaluations. Most importantly, the authors were limited by the description of the ulcer in the medical record. Characteristics of the ulcer, such as depth, may not be well standardized in a large group of practitioners who are not ophthalmologists. In some cases, data in the medical records could also be missing; however, the large number of cases does provide a more robust result despite the study's retrospective nature. The descriptions associated with corneal ulceration, such as allergic and uveitis, are not likely to be causes of the ulceration. They are important findings that could relate to the development of ulceration (e.g., allergic skin disease resulting in rubbing and ulceration) but do not directly cause the ulceration. Similarly, uveitis is unlikely to be the cause of the ulceration but could be a secondary development. Neoplasia (e.g., an eyelid mass) could be considered a more direct cause of ulceration. Another limitation of this study is the lack of follow‐up for many of the cases. Most cases were evaluated and treated locally, but it is possible that a horse could have moved or switched to another veterinarian. This bias would primarily affect the description of outcomes in the case, and the actual rate of enucleation or euthanasia may have been higher.

As with all retrospective studies, distinguishing between an association and a cause is important. Although a number of interesting associations were found between the frequency of corneal ulcers and the frequency of enucleation, the hypothesis‐generating nature of this study cannot test causality. Additional prospective studies are needed to further evaluate the risk factors.

In conclusion, corneal ulceration in this regional study was more common in male horses and occurred more frequently in times with increased wind speed and temperatures. Deeper corneal ulcers, those involving the left eye, the age of the horse, and the presence of uveitis were factors associated with an increased risk of enucleation.

## Author Contributions

**Valeria Adamchick Galindo**: conceptualization, investigation, writing – original draft, methodology, writing – review and editing, formal analysis, visualization. **Langdon Fielding**: conceptualization, investigation, methodology, validation, writing – review and editing, software, formal analysis, project administration, supervision, visualization, data curation.

## Conflicts of Interest

The authors declare no conflicts of interest.

## Offprints

Offprints will not be available.

## Data Availability

The data that support the findings of this study are available on request from the corresponding author. The data are not publicly available due to privacy or ethical restrictions.

## Endnotes

1 GraphPad Prism version 10.0.2 for Windows, GraphPad Software, La Jolla, California.
